# Supplementary material for: A Multivariate Genome-Wide Association Analysis of 10 LDL Subfractions, and Their Response to Statin Treatment, in 1868 Caucasians
Source: PLoS One. 2015 Apr 21;10(4):e0120758. doi: 10.1371/journal.pone.0120758 (PMC4405269; doi:10.1371/journal.pone.0120758)
Supplement: S1 Text — (PDF) [file pone.0120758.s001.pdf]

Computing proportion of variance in phenotype explained by a given SNP (PVE)

[1] provides sample size, minor allele frequency (MAF), effect size, and standard error of effect size for each reported SNP (see Supplementary Table 2 in [1]). We estimated PVE using the information from [1] as follows. Variance in phenotype (Y) can be decomposed into two components:

$$\text{Var}(Y) = \beta^2\text{Var}(X) + \sigma^2, \tag{1}$$

where β is effect size of genetic variant (X). The first component ($\beta^2\text{Var}(X)$) captures variance explained by the genetic variant X and the second component (σ^2) captures the remaining variance that can be explained by environmental factors or other genetic variants. We can estimate $\beta^2\text{Var}(X)$ by $2\hat{\beta}^2$MAF (1-MAF), where $\hat{\beta}$ and MAF are effect size estimate and minor allele frequency for the genetic variant X, respectively. From a simple linear regression model (X and Y as covariate and response),

$$\text{Var}(\hat{\beta}) = (\text{se}(\hat{\beta}))^2 \approx \frac{\sigma^2}{2NMAF(1-MAF)}, \tag{2}$$

where N is sample size and se($\hat{\beta}$) is standard error of effect size for the genetic variant X. Therefore,

$$PVE = \frac{\beta^2\text{Var}(X)}{\text{Var}(Y)} = \frac{\beta^2\text{Var}(X)}{\beta^2\text{Var}(X) + \sigma^2} \tag{3}$$

can be estimated by

$$\frac{2\hat{\beta}^2MAF(1-MAF)}{2\hat{\beta}^2MAF(1-MAF) + (\text{se}(\hat{\beta}))^2 2NMAF(1-MAF)}. \tag{4}$$

We compute PVE for HDL-C (LDL-C) by using the information for the most strongly associated SNP rs3764261 (rs247616). Note that rs3764261 is in high LD ($r^2 = 0.96$) with rs247616.

References

1. Teslovich TM, Musunuru K, Smith AV, Edmondson AC, Stylianou IM, Koseki M, et al. Biological, clinical and population relevance of 95 loci for blood lipids. Nature. 2010;466:707–713.
