# Supplementary figures and images for: A Multivariate Genome-Wide Association Analysis of 10 LDL Subfractions, and Their Response to Statin Treatment, in 1868 Caucasians
Source: PLoS One. 2015 Apr 21;10(4):e0120758. doi: 10.1371/journal.pone.0120758 (PMC4405269; doi:10.1371/journal.pone.0120758)

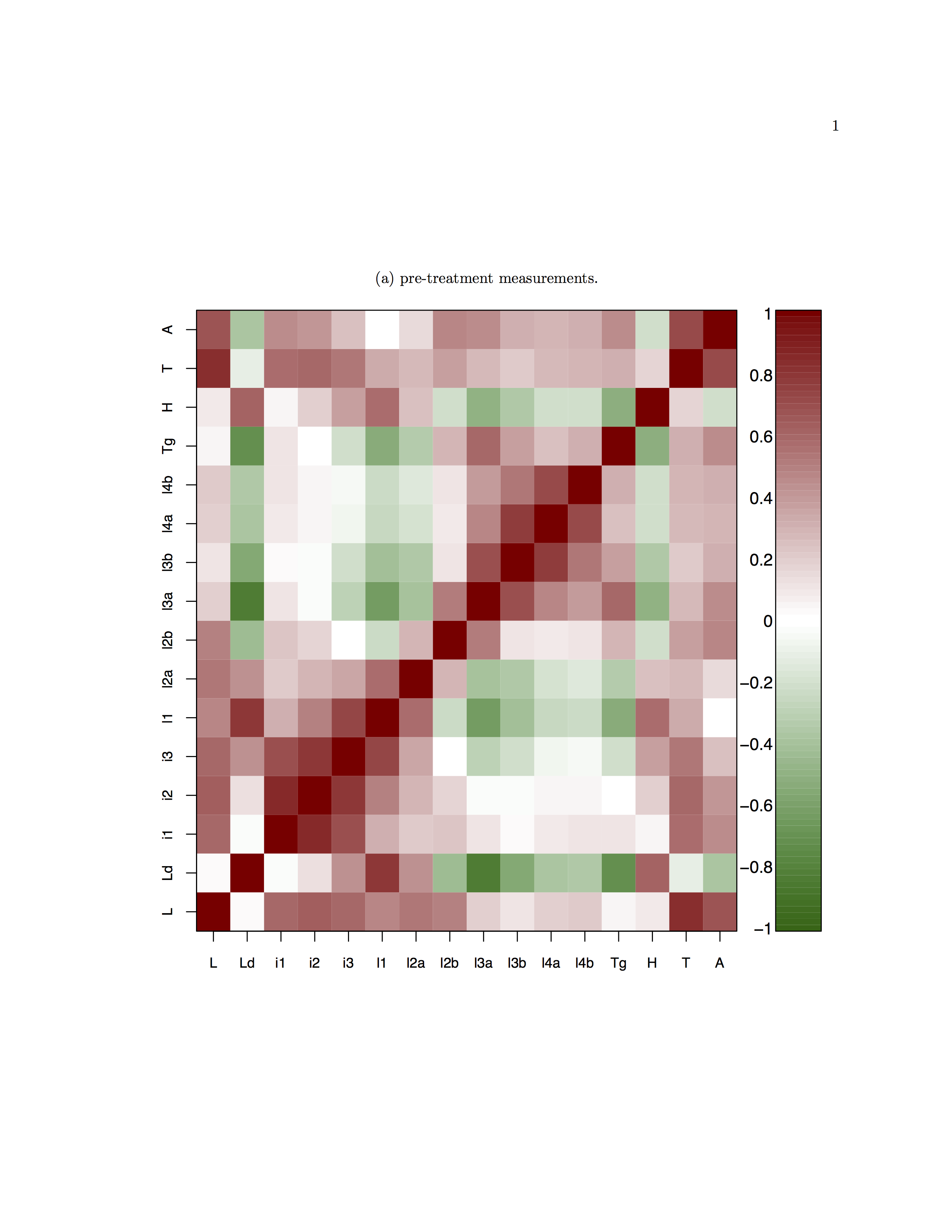

Supplement: S1 Fig — In addition to 12 phenotypes considered in our main analysis, correlations with triglycerides (Tg), HDL-cholesterol (H), total cholesterol (T), ApoB levels (A) are also included. See Table 1 in the main text for abbrevations for 12 phenotypes. Correlations are computed by using (a) (normalized) pre-treatment measurements (P in the methods section); (b) (normalized) post-treatment measurements (T in the methods); (c) (normalized) averages of pre-treatment measurements and post-treatment measurements (A˜ in the methods); (d) (normalized) differences between post-treatment and pre-treatment measures (D˜ in the methods). (TIFF) [file pone.0120758.s004.tiff]

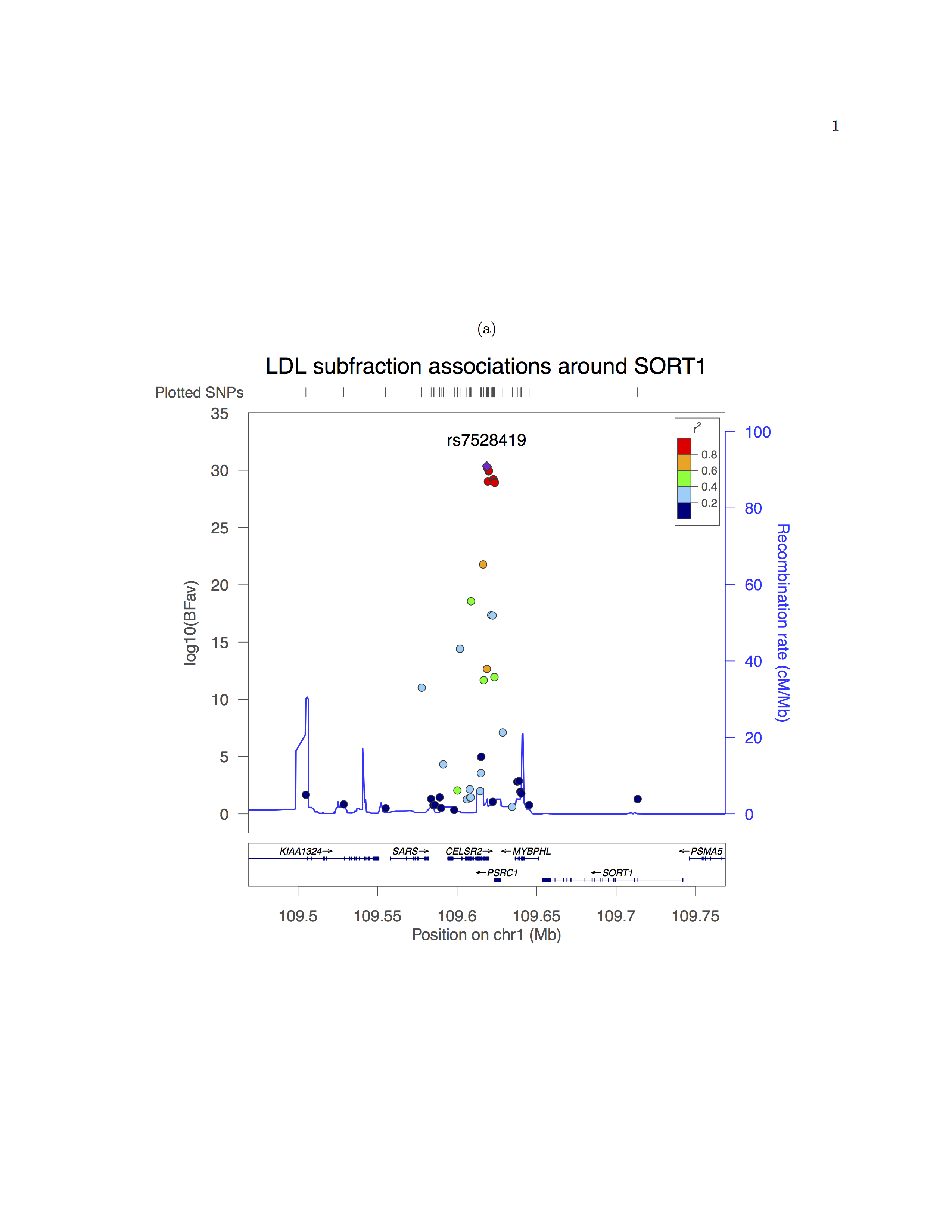

Supplement: S2 Fig — Regional plots of the four loci are created by using the tool LocusZoom [43] (with log10 BFav in y-axis). Purple diamond is the top SNP with the strongest association in each locus. Each circle corresponds to a SNP whose color indicates the linkage disequilibrium with the top SNP. LDL subfraction associations around (a) SORT1, (b) APOE, (c) LPA, and (d) CETP. (TIFF) [file pone.0120758.s005.tiff]

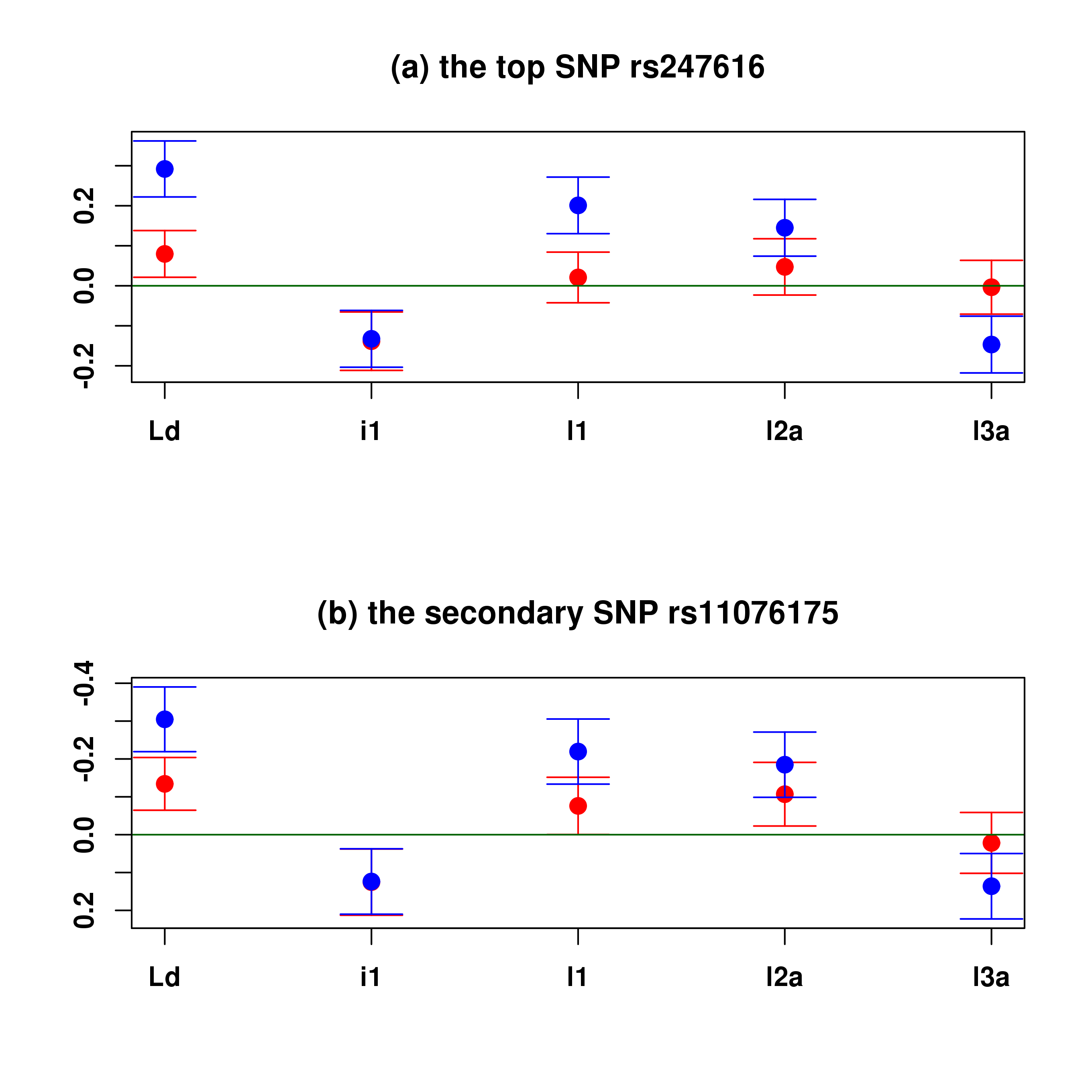

Supplement: S3 Fig — (a) The top SNP rs247616 and (b) the secondary SNP rs11076175 in CETP. Note that, because the minor alleles have opposite effects on total HDL-C at the two SNPs, the y-axis is reversed for the secondary SNP to emphasize the similar pattern of effect size. (TIFF) [file pone.0120758.s006.tiff]
