# Supplementary material for: A Multivariate Genome-Wide Association Analysis of 10 LDL Subfractions, and Their Response to Statin Treatment, in 1868 Caucasians
Source: PLoS One. 2015 Apr 21;10(4):e0120758. doi: 10.1371/journal.pone.0120758 (PMC4405269; doi:10.1371/journal.pone.0120758)
Supplement: S1 Table — (PDF) [file pone.0120758.s007.pdf]

S1 Table: Summary of the top and secondary associations with LDL subfractions in our analysis.

Gene	SORT1	APOE	APOE	LPA	LPA	CETP	CETP
chr	1	19	19	6	6	16	16
type	top	top	secondary[1]	top	secondary	top	secondary
SNP	rs7528419	rs7412	rs157581	rs55730499	6-161069320	rs247616	rs11076175
LDL-C (L)							
$\log_{10}$ BF[2]	3.07	7.18	3.62	-0.28	-0.44	-0.12	-0.05
PP of direct association[3]	0.95	0	0.96	0.07	0.06	0.15	0.27
PP of indirect association[3]	0.05	1	0.04	0.03	0.12	0.41	0.63
PP of no association[3]	0	0	0	0.9	0.82	0.44	0.1
LDL peak particle diameter (Ld)							
$\log_{10}$ BF	-0.48	-0.08	-0.36	-0.28	-0.41	13.39	3.24
PP of direct association	0	0.7	0.41	0.01	0.07	0	0.98
PP of indirect association	0.13	0.23	0.45	0.03	0.17	1	0.02
PP of no association	0.86	0.07	0.14	0.96	0.76	0	0
IDL1 (i1)							
$\log_{10}$ BF	-0.20	1.47	0.2	5.25	1.17	2.06	-0.01
PP of direct association	0.74	0.01	0.55	0	1	0.6	0.31
PP of indirect association	0.03	0.98	0.35	1	0	0.4	0.58
PP of no association	0.23	0.01	0.1	0	0	0	0.1
IDL2 (i2)							
$\log_{10}$ BF	-0.06	0.22	-0.25	-0.09	-0.26	0.16	-0.39
PP of direct association	0.76	0.22	0.61	0	0.15	0.65	0.27
PP of indirect association	0.03	0.7	0.12	0.06	0.21	0.29	0.47
PP of no association	0.21	0.09	0.28	0.94	0.63	0.06	0.26
IDL3 (i3)							
$\log_{10}$ BF	-0.12	-0.32	0.01	-0.24	-0.46	-0.38	-0.24
PP of direct association	0.76	0.61	0.45	0	0.11	0.31	0.19
PP of indirect association	0.03	0.24	0.43	0.03	0.01	0.23	0.69
PP of no association	0.21	0.15	0.12	0.97	0.88	0.46	0.12
LDL1 (l1)							
$\log_{10}$ BF	-0.31	1.69	0.97	-0.29	-0.46	5.75	1.42
PP of direct association	0.75	0.71	0.45	0.07	0.07	0.86	0.21
PP of indirect association	0.03	0.29	0.54	0.03	0.08	0.14	0.78
PP of no association	0.22	0	0.01	0.9	0.85	0	0.01
LDL2a (l2a)							
$\log_{10}$ BF	-0.41	5.02	1.08	-0.29	-0.43	2.60	1.06
PP of direct association	0.71	0.74	0.39	0.06	0.06	0.86	0.21
PP of indirect association	0.03	0.26	0.59	0.03	0.16	0.14	0.77
PP of no association	0.26	0	0.02	0.91	0.78	0	0.02
LDL2b (l2b)							
$\log_{10}$ BF	-0.12	2.51	-0.08	-0.26	-0.45	-0.31	-0.43
PP of direct association	0.68	0.39	0.53	0.06	0.05	0.79	0.25
PP of indirect association	0.05	0.61	0.28	0.03	0.15	0.12	0.39
PP of no association	0.27	0	0.2	0.91	0.8	0.09	0.36
LDL3a (l3a)							
$\log_{10}$ BF	1.32	0.63	-0.15	-0.29	-0.46	2.68	0.08
PP of direct association	0.97	0.68	0.36	0.08	0.05	0.81	0.22
PP of indirect association	0.03	0.31	0.54	0.03	0.15	0.19	0.73
PP of no association	0	0.01	0.1	0.89	0.8	0	0.05
LDL3b (l3b)							
$\log_{10}$ BF	11.84	0.41	0.30	-0.29	-0.45	0.45	-0.40
PP of direct association	0.94	0.54	0.41	0.06	0.05	0.87	0.19
PP of indirect association	0.06	0.44	0.54	0.03	0.16	0.13	0.62
PP of no association	0	0.02	0.05	0.91	0.79	0.01	0.19
LDL4a (l4a)							
$\log_{10}$ BF	28.92	-0.13	1.52	-0.29	-0.38	-0.52	-0.44
PP of direct association	0	0.54	0.63	0.09	0.06	0.62	0.21
PP of indirect association	1	0.37	0.35	0.03	0.15	0.13	0.54
PP of no association	0	0.09	0.02	0.88	0.79	0.25	0.25
LDL4b (l4b)							
$\log_{10}$ BF	12.99	-0.14	0.86	-0.28	-0.31	-0.53	-0.44
PP of direct association	0.97	0	0.4	0.09	0.06	0.16	0.26
PP of indirect association	0.03	0.71	0.57	0.03	0.16	0.26	0.4
PP of no association	0	0.28	0.03	0.88	0.78	0.59	0.35

Abbreviations for each phenotype are in parentheses.
[1] Results for secondary SNPs are from analysis on the residuals obtained by regressing out the effects of the most strongly associated SNP (top SNP) in each gene.
[2] $\log_{10}$Bayes Factor from a univariate analysis of each phenotype.
[3] Marginal posterior probability of direct, indirect, no association for each phenotype that is computed from our multivariate analysis. Here, we compute these posterior probabilities conditional on at least one phenotype being directly associated.
